# The Giving Voice to Mothers study: inequity and mistreatment during pregnancy and childbirth in the United States

**DOI:** 10.1186/s12978-019-0729-2

**Published:** 2019-06-11

**Authors:** Saraswathi Vedam, Kathrin Stoll, Tanya Khemet Taiwo, Nicholas Rubashkin, Melissa Cheyney, Nan Strauss, Monica McLemore, Micaela Cadena, Elizabeth Nethery, Eleanor Rushton, Laura Schummers, Eugene Declercq, the GVtM-US Steering Council

**Affiliations:** 1 Birth Place Lab, Division of Midwifery, Faculty of Medicine, University of British Columbia, Vancouver (Canada), E416 Shaughnessy (Mailbox 80), 4500 Oak Street, Vancouver, BC V6H 3N1 Canada; 2 University of California Davis School of Medicine, Sacramento, California, USA; 3 Department of Midwifery, Bastyr University, Seattle, WA USA; 4 Department of Obstetrics and Gynecology, University of California San Francisco and the Institute for Global Health Sciences, California, USA; 5 Department of Anthropology, Oregon State University, Corvallis, Oregon, USA; 6 Every Mother Counts, New York City, USA; 7 Department of Family Health Care Nursing and ANSIRH Bixby Center for Global Reproductive Health, University of California, San Francisco, USA; 8 Young Women United, Albuquerque, New Mexico, USA; 9 School of Population & Public Health, Faculty of Medicine, University of British Columbia, Vancouver, Canada; 10 Department of Family Practice, Faculty of Medicine, University of British Columbia, Vancouver, Canada; 11 School of Public Health, Boston University, Massachusetts, Boston, USA

**Keywords:** Respectful maternity care, Mistreatment, Pregnancy, Childbirth, Race, Disrespect, Abuse, Participatory research, Hospital birth, Home birth, Health equity, Midwifery, Quality measure

## Abstract

**Background:**

Recently WHO researchers described seven dimensions of mistreatment in maternity care that have adverse impacts on quality and safety. Applying the WHO framework for quality care, service users partnered with NGOs, clinicians, and researchers, to design and conduct the Giving Voice to Mothers (GVtM)–US study.

**Methods:**

Our multi-stakeholder team distributed an online cross-sectional survey to capture lived experiences of maternity care in diverse populations. Patient-designed items included indicators of verbal and physical abuse, autonomy, discrimination, failure to meet professional standards of care, poor rapport with providers, and poor conditions in the health system. We quantified the prevalence of mistreatment by race, socio-demographics, mode of birth, place of birth, and context of care, and describe the intersectional relationships between these variables.

**Results:**

Of eligible participants (*n* = 2700), 2138 completed all sections of the survey. One in six women (17.3%) reported experiencing one or more types of mistreatment such as: loss of autonomy; being shouted at, scolded, or threatened; and being ignored, refused, or receiving no response to requests for help. Context of care (e.g. mode of birth; transfer; difference of opinion) correlated with increased reports of mistreatment. Experiences of mistreatment differed significantly by place of birth: 5.1% of women who gave birth at home versus 28.1% of women who gave birth at the hospital. Factors associated with a lower likelihood of mistreatment included having a vaginal birth, a community birth, a midwife, and being white, multiparous, and older than 30 years.

Rates of mistreatment for women of colour were consistently higher even when examining interactions between race and other maternal characteristics. For example, 27.2% of women of colour with low SES reported any mistreatment versus 18.7% of white women with low SES. Regardless of maternal race, having a partner who was Black also increased reported mistreatment.

**Conclusion:**

This is the first study to use indicators developed by service users to describe mistreatment in childbirth in the US. Our findings suggest that mistreatment is experienced more frequently by women of colour, when birth occurs in hospitals, and among those with social, economic or health challenges. Mistreatment is exacerbated by unexpected obstetric interventions, and by patient-provider disagreements.

**Electronic supplementary material:**

The online version of this article (10.1186/s12978-019-0729-2) contains supplementary material, which is available to authorized users.

## Plain English summary

Global health experts agree that how people are treated during childbirth can affect the health and well-being of mother, child, and family, but very little is known about experiences of care among childbearing populations in the United States. In this study, community members worked with researchers to design a survey that would capture their lived experiences of care during pregnancy and childbirth, including seven types of mistreatment by health providers or health systems. We collected information across the country including from communities of colour, and women who planned to give birth at home or in a birthing center. Of the 2700 women who filled out the survey, one in six (17.3%) reported mistreatment. Among all participants, being shouted at or scolded by a health care provider was the most commonly reported type of mistreatment (8.5%), followed by “health care providers ignoring women, refusing their request for help, or failing to respond to requests for help in a reasonable amount of time” (7.8%). Some women reported violations of physical privacy (5.5%), and health care providers threatening to withhold treatment or forcing them to accept treatment they did not want (4.5%). Women of colour, women who gave birth in hospitals, and those who face social, economic, or health challenges reported higher rates of mistreatment. Rates were also increased in women who had unexpected events like cesareans or transfer from community to hospital care; and women who disagreed with a health care provider, about the right care for themselves or the baby, reported the highest rates of mistreatment.

## Background

High quality, respectful maternity care is a global priority [1]. In 2017, the World Health Organization (WHO) published eight standards for quality of maternal and newborn care that can be used to evaluate “the extent to which health care services provided to individuals and patient populations improve desired health outcomes and [are] safe, effective, timely, efficient, equitable and people-centred” [2]. Four of the standards emphasize care that demonstrates respect, dignity, emotional support, and a systemic commitment to a patient-led, informed decision-making process. The International Federation of Gynecologists and Obstetrics, the International Confederation of Midwives, the International Pediatric Association, and the White Ribbon Alliance have prioritized the WHO quality care standards, and protection of human rights in childbirth, as essential to optimizing birth outcomes [3].

Care provider actions and interactions are associated with women’s experience of trauma during birth, as indicated in an online survey (*n* = 748) [4]. Qualitative analysis identified four common themes: ‘prioritizing the care provider’s agenda’; ‘disregarding embodied knowledge’; ‘lies and threats’; and ‘violation’ [4]. A traumatic birth can have serious impact on postnatal mental health and family relationships. Short-term consequences of adverse experience of care include pain and suffering, and long-term consequences cited in the international literature include post-traumatic stress disorder, fear of birth, negative body image, and feelings of dehumanization [4–7]. In addition to these outcomes, fear of disrespect and abuse, and loss of autonomy have been cited as drivers for planned unattended home births, and reduce uptake of care, even among women with known risk factors [8]. Indeed, such mistreatment is itself an adverse outcome as it constitutes a violation of basic human rights [9].

Recognizing these serious health impacts, the World Health Organization (WHO) issued a statement in 2014 calling for further research on defining and measuring disrespect and abuse in public and private facilities worldwide [10, 11]; and urged health systems to protect and promote women’s rights to dignified and respectful care, in addition to ensuring universal access to timely, safe and effective clinical care [11]. While significant disparities in maternal and newborn outcomes are reported across populations in the United States (US) [12], very little is known about whether mistreatment is a component of these adverse outcomes. To understand experiences of childbirth care, especially among communities of color and those who choose to deliver in community settings, service users partnered with NGOs, clinicians, and researchers, to conduct the Giving Voice to Mothers (GVtM)–US study.

### Measuring mistreatment in high resource countries

To date, evaluations of respectful maternity care (RMC) have focused primarily on monitoring care during hospital births in low-resource settings [6, 13, 14]. However, childbearing women from high and middle resource countries have also reported negative experiences during hospital births, including being ignored, belittled or verbally humiliated by healthcare providers, having interventions forced upon them, and being separated from their babies without reason or explanation [7, 15–17]. For example, women from Slovakia who were interviewed (*n* = 15) reported that care providers treated them as objects incapable of making decisions about their own care. Many of them did not consent to interventions such as episiotomies. Violations of their dignity, privacy, and confidentiality were common. Women said that care providers did not listen to them, doubted their perceptions and feelings, ignored their wishes, imposed their will on women, and made them feel guilty or like failures [17].

In high resource countries, pregnant people who are recent immigrants, Indigenous, and/or disenfranchised by their lower socioeconomic status, race/ethnicity, incarceration, substance dependence, or housing instability have been reported to be at increased risk for poor health outcomes, and reduced access to high quality care [18–22]. Few investigators have examined whether experiences of RMC differ by sociodemographic factors, but one U.S. national study identified racial disparities in the treatment of childbearing women in hospitals [23]. Among respondents, 30% of Black and Hispanic primiparous women and 21% of White women who delivered in hospitals in the US reported that they were “treated poorly because of a difference of opinion with [their] caregivers about the right care for [herself or her] baby” [23].

In 2015, the WHO Research Group on Treatment of Women During Childbirth conducted a systematic review of the literature on RMC [13]. Bohren and colleagues examined qualitative and quantitative evidence from 65 studies on the mistreatment of women during childbirth in health facilities across 34 countries, representing diverse geographical and economic settings. The investigators identified multiple examples of disrespect and human rights violations experienced by women giving birth, ranging from physical and verbal abuse, to a lack of supportive care, to neglect, discrimination, and denial of autonomy [13]. Noting wide inconsistencies in terminology and definitions of disrespect and abuse, the authors named the phenomenon “mistreatment” and delineated the phenomena across seven dimensions: physical abuse, sexual abuse, verbal abuse, stigma and discrimination, failure to meet professional standards of care, poor rapport between women and providers, and poor conditions and constraints presented by the health system [13]. They proposed that future investigators utilize this typology to inform studies that seek to understand the prevalence and impact of mistreatment across jurisdictions or populations, and/or to evaluate the success of interventions. Since 2015, numerous authors have responded to the Bohren typology, noting a lack of global evidence on the topic [24–27]. Some investigators have adapted the typology to qualitative studies of the prevalence and characteristics of mistreatment in low resource countries [14], but none to date have applied the typology to assess experience of care in high resource countries, and none have assessed the seven domains in a quantitative survey.

Notably, while the lived experience among study participants provided the descriptive data that informed the Bohren typology, none of the studies included in the systematic review used a patient-led approach to item development. Best practice in patient-oriented outcomes research would suggest that “mistreatment” as an outcome may be best described and delineated by the recipients of care. Patient experience indicators of quality and safety are now routinely collected at institutions in other areas of medicine, yet patient-designed instruments that can assess the impact of experience of maternity care remain scarce.

In this paper, we introduce a set of patient-designed indicators of mistreatment that align with the typology proposed by Bohren et al., and are relevant to service users in high resource settings. We present results from a large national survey that utilized these items to examine how women in the US overall, and among key subgroups, report on mistreatment during pregnancy and childbirth. In addition, we examine the relationships between race and mistreatment in the context of factors that are frequently related to health inequity. The concept of intersectionality is rarely considered during design, analysis or interpretation of public health studies [28]; we aimed to address this gap in this study.

## Methods

In 2016, using a community-based participatory research process [29, 30], we convened a multi-stakeholder team to launch Giving Voice to Mothers (GVtM-US), a study of maternity care experiences of women who experienced pregnancy in the United States between 2010 and 2016. The only previous national study on experience of maternity care in the US was limited to women who planned hospital births, had limited information on differential experiences by race, and did not measure mistreatment [23]. Hence, our team, comprised of community members, clinicians, community health service leaders, and researchers designed a study on quality of maternity care as experienced by pregnant persons from 4 communities of colour (African American, Indigenous, Hispanic, and Asian) who gave birth in any location, as well as women who planned to give birth in homes and freestanding birth centers. The Behavioural Research Ethics Board at University of British Columbia approved the study (H15–01524). All participants reviewed an informed consent form before deciding whether they wanted to participate in the online survey.

### Survey development

The GVtM Steering Council recruited community agency leaders and service providers to adapt a survey instrument, developed by service users to study maternity care experiences in British Columbia, Canada [31–33], to the United States context. The validated instrument explored four domains including: preferences for care, interactions with care providers, role in decision-making, and access to care options. Following consultations with the communities they serve, the GVtM Steering Council identified, drafted, or adapted additional items from the literature that assess non-consensual care, disparities in access, social determinants, and institutional racism [34, 35]. Some items had been used to measure disrespect and abuse in low resource countries and were adapted for application to the US context [35].

The community agencies (NGOs) then recruited 57 women from the target populations to review the draft, and subsequently 31 community members, with representation from all target populations, served on an expert panel to formally content validate the adapted instrument. They rated each item on a 4-point ordinal scale for clarity, relevance, and importance and provided narrative commentary. We retained, revised, or discarded items based on best practice guidelines for content validation [36]. The community members strongly endorsed the inclusion of the previously validated quality measures, the Mothers Autonomy in Decision Making (MADM) scale [31] and the Mothers on Respect (MOR) index [32]. They also adapted the Perceptions of Racism (PR) scale [34] to be inclusive of all study populations. Community members suggested inclusion of additional novel items in the instrument such as “*When you experience problems, what helps you and your family survive, succeed and thrive?”* and, in cases of refusal of care, *“How did your doctor or midwife react?”* and *“Who stood up for you?”*. They provided detailed answer options that reflected their lived experience.

Most questions had pre-defined Likert response options, but the survey instrument also included several open-ended questions to allow participants to provide explanatory detail. The final GVtM survey instrument contained 218 items (the full list of survey items is available upon request via: http://www.birthplacelab.org/contact-us/), with 60 items measuring aspects of mistreatment. It was translated and back translated into a Spanish version, and both versions were mounted on an online platform that allowed for branching to questions adapted for participants who experienced pregnancy loss, and for those who were currently pregnant.

### Inclusion criteria

**Fig. 1:**
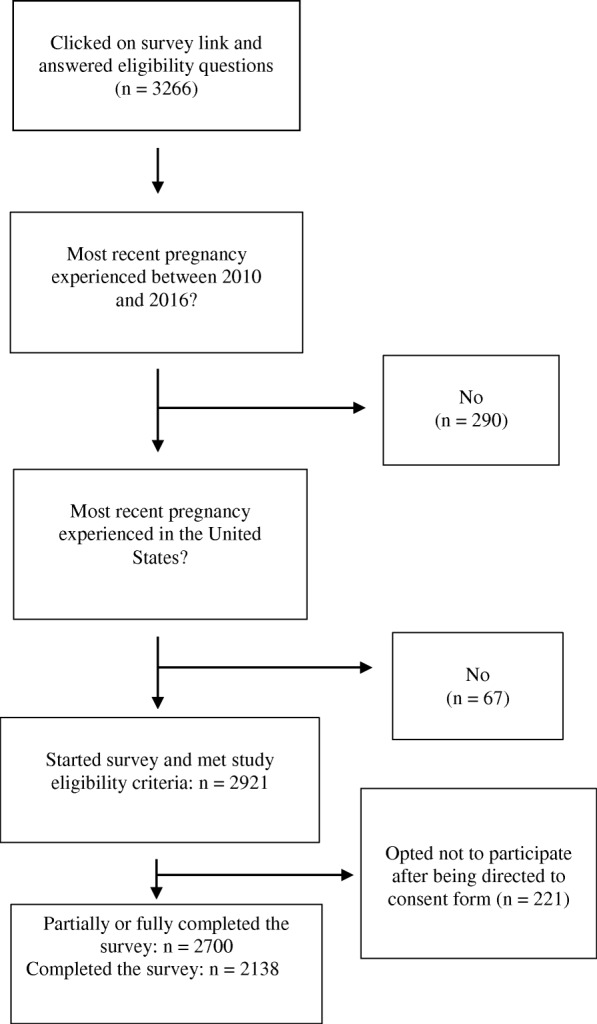
Sample Size Flow Chart

Women who experienced at least one pregnancy in the United States between 2010 and 2016, including those currently pregnant, could participate. Of the 2700 women who completed or partially completed the survey, some participants skipped questions and others did not finish the survey, resulting in variable denominators for each section. Because we compare variables that appear across the entire survey, we restrict our analysis to the 2138 women who completed the survey. Details on sample delineation are in Fig. 1.

### Recruitment

All partners participated in evidence-based strategies for recruitment of traditionally marginalized groups, including social networking and venue-based sampling [37–39]. We used strategies to ensure strong representation of women of colour, and women who planned a birth at home or at a freestanding birthing center. For example, we engaged agencies in study recruitment who serve these populations, and some held survey café events with computer access available, and/or trained peers, known as “data doulas” [40] to support participants with their own data entry. To achieve our goal of robust sampling from women of colour and those who chose home and birth center births, based on the rates of participation to date, halfway through the data collection period we closed the survey to women who identified as White and who gave birth in a hospital, but kept it open to other participants.

In New York State data collection was embedded in an established ongoing statewide maternity care evaluation project led by one of the NGO partners, Choices in Childbirth. The Steering Council recognized that this was likely to lead to oversampling from a single state; hence, they initially considered launching the study as a New York State pilot study to demonstrate feasibility and generate enough data to highlight need for national follow up. However, community members served by the distributed NGOs and clinicians on the team felt strongly that they wanted the GVtM study to be open to participants from rural, urban, and suburban contexts *across* the United States. They felt that social media recruitment had the greatest potential for securing comparative data from a wide range of service users. Hence, to respect an authentic, patient-oriented participatory research process, the survey was distributed nationally. The GVtM survey was open from March 2016–March 2017.

### Measurement

#### Mistreatment

Content validation resulted in new patient-designed and patient-validated items to measure mistreatment in childbirth that align with the dimensions codified by Bohren (Table 1) [13]. Of note, the community members on the Steering Council and the women who participated during the expert content validation stage endorsed these items without knowledge of the Bohren systematic review in progress, yet their lived experience resonated with the typology. Specifically, the mistreatment items measure the following domains: physical abuse, sexual abuse, verbal abuse, neglect and abandonment, poor rapport between women and providers, loss of confidentiality, and lack of supportive care. Community members also elected to include the MADM (autonomy) and MOR (respect), and an adapted Perceptions of Racism scale [34] that measure other domains in the Bohren typology: stigma and discrimination, failure to meet professional standards of care, lack of informed consent, and loss of autonomy. Twenty-two additional survey items related to the typology and assessed RMC, such as care provider behaviors in response to refusal of care, and the respondent’s overall sense of dignity, respect, and privacy during interactions with providers.

**Table 1:** GVtM items that align with WHO [63] typology of mistreatment

Bohren et al. – Third-Order Themes	Bohren et al. – Second -Order Themes	GVtM – US items and scales
Physical abuse	Use of force	“You experienced physical abuse (including aggressive physical contact, inappropriate sexual conduct, a refusal to provide anesthesia for an episiotomy, etc.)”
	Physical restraint	
Sexual abuse	Sexual abuse	
Verbal abuse	Harsh language	“Health care providers (doctors, midwives, or nurses) shouted at or scolded you”
	Threats and blaming	“Health care providers threatened to withhold treatment or to force you to accept treatment you did not want”
		“Health care providers threatened you in any other way”
Stigma and discrimination	Discrimination based on socio-demographic characteristics	Mothers on Respect (MOR) Index (14 items)^a^
		• Adapted 17-item Perceptions of Racism Scale
		• Four items that assess perceived discrimination from care providers or other disrespectful care provider behaviours, e.g. During my pregnancy I held back from asking questions or discussing my concerns because I felt discriminated against; During my pregnancy I held back from asking questions or discussing my concerns because my care provider used language I could not understand.
		• One item asking women how often they have felt treated unfairly because of their race, heritage or ethnic group
Failure to meet professional standards of care	Lack of informed consent and confidentiality	“Your private or personal information was shared without your consent”
		“Your physical privacy was violated (i.e., being uncovered or having people in the delivery room without your consent)”
	Physical examinations and procedures	“My doctor or midwife explained different options for care during my labour and birth.”
		“My doctor or midwife asked me what I wanted to do before the following procedures were done: (episiotomy, continuous fetal monitoring, screening tests etc).”
	Neglect and abandonment	
		“Health care providers ignored you, refused your requests for help, or failed to respond to requests for help in a reasonable amount of time.”
Poor rapport between women and providers	Ineffective communication	Mother Autonomy in Decision Making scale (MADM) (7 items)^b^
		• Three items that ask women to rate the level of respect, dignity and privacy that their care provider showed during labour and/or birth
	Lack of supportive care	
		• Five items about care that women declined, what they declined, why, how their care provider reacted and if anyone helped the woman maintain her wishes.
	Loss of autonomy	
Health system conditions and constraints	Lack of policies	Adapted Perceptions of Racism Scale included items assessing treatment in medical offices and hospital wards
	Facility culture	

^a^Vedam S, Stoll K, Rubashkin N, et al. The Mothers on Respect (MOR) index: measuring quality, safety, and human rights in childbirth. Social Science and Medicine: Population Health. 10.1016/j.ssmph.2017.01.005

^b^Vedam S, Stoll K, Martin K, et al. The Mother’s Autonomy in Decision Making (MADM) scale: Patient-led development and psychometric testing of a new instrument to evaluate experience of maternity care. PLOS ONE 10.1371/journal.pone.0171804

The focus of the current paper is application of mistreatment items that describe patient experience of provider behaviors. Subsequent reports will focus on analysis of data related to the mistreatment domains of autonomy and respect (eg. MADM, MOR, and PR scale scores), and non-consented care among the GVtM participants.

#### Maternal/paternal race

Community members on the study team recommended that research that relies on US Census categories fails to capture the lived experience of people who self-identify across more than one race, and/or experience the effects of visible minority race. Accordingly, the team designed a complex but respectful and realistic approach to collecting and coding this set of items. Respondents could self-identify and provide considerable detail about their identity, selecting multiple descriptors under 13 pre-defined categories. For analysis, we recoded this variable into mutually exclusive categories (see Additional file 1: Table S1). We used the same coding scheme for paternal race/ethnic identity (as identified by the woman), and also created four variables that describe combinations of maternal/paternal race, i.e. 1) woman white, partner white, 2) women black, partner black, 3) women white, partner black, 4) women black, partner white. Throughout this paper Indigenous includes participants who self-identify as Native American, Native Hawaiian or Pacific Islander, Alaska Native, or Indigenous to Mexico or South America.

#### Low SES

We created a comprehensive composite index that measures low SES, taking into account family income below the federal poverty threshold (based on before tax family income and household size). In the low SES category, we also counted respondents who reported that their heat or electricity was turned off (during or in the year before pregnancy), inability to buy enough food or meet financial obligations; and respondents who reported receiving a housing subsidy, assistance from Indian Health Services or a state health plan, Temporary Assistance for Needy Families (TANF), food stamps, WIC food vouchers or money to buy food. We coded respondents with one or more of the indicators of low SES as 1; and respondents that did not report any of the indicators as 0.

#### History of social risks

To distinguish those who may experience differential treatment because of social factors, we grouped together respondents who reported substance use (smoking, daily alchohol use during pregnancy, and/or drug dependence) during pregnancy, women with a history of incarceration (herself or partner), involvement of child or family services, and/or intimate partner violence. Women who reported one or more of the indicators of social risk were coded as 1; women did not report any social risk indicators were coded as 0. We also created composite indices that measure elevated pregnancy risks and newborn health problems. A description about how these indices were derived can be found in footnotes below the tables.

### Analysis

To describe the overall prevalence of mistreatment in the study population, we calculated the proportion of women who experienced each of the seven types of mistreatment and what proportion experienced any mistreatment (i.e. any of the seven indicators). We report sociodemographic variables for all women who started the survey and met eligibility criteria (*n* = 2700), as well as for all women who completed the last item on the survey (*n* = 2138). Rates of mistreatment are stratified by maternal characteristics such as race, parity, age, immigrant status, SES, pregnancy health status, and social risks (history of substance use, incarceration and/or intimate partner violence); as well as context of care factors (induction, mode of birth, place of birth, type of provider, and disarticulation between their own preferences for care and their provider’s recommendations). We used logistic regression to quantify the relationship between mistreatment and the variables described above. To examine the relationship between mistreatment and maternal race/ethnicity, we calculated odds ratios comparing the odds of mistreatment among women of color to the odds among white women.

To elucidate the intersectional relationships between maternal race and other factors that are linked to mistreatment, we examined the relationship between race and mistreatment within categories of other sociodemographic and context of care variables. Within categories (e.g., nulliparous, age 17–25 years, place of birth), we calculated the prevalence of mistreatment among women of colour and white women separately. Larger differences between groups indicate larger disparities in mistreatment by race.

To report illustrative details provided in open-ended text boxes, community and research team members verified the applicability and resonance of the Bohren framework and recommended that we include the voices of mothers by identifying exemplars based on the Bohren typology. Three team members independently reviewed the text boxes and came to consensus about representative quotes, which were then reviewed and approved by the community partners.

## Results

### Sample (*n* = 2138)

**Fig. 2:**
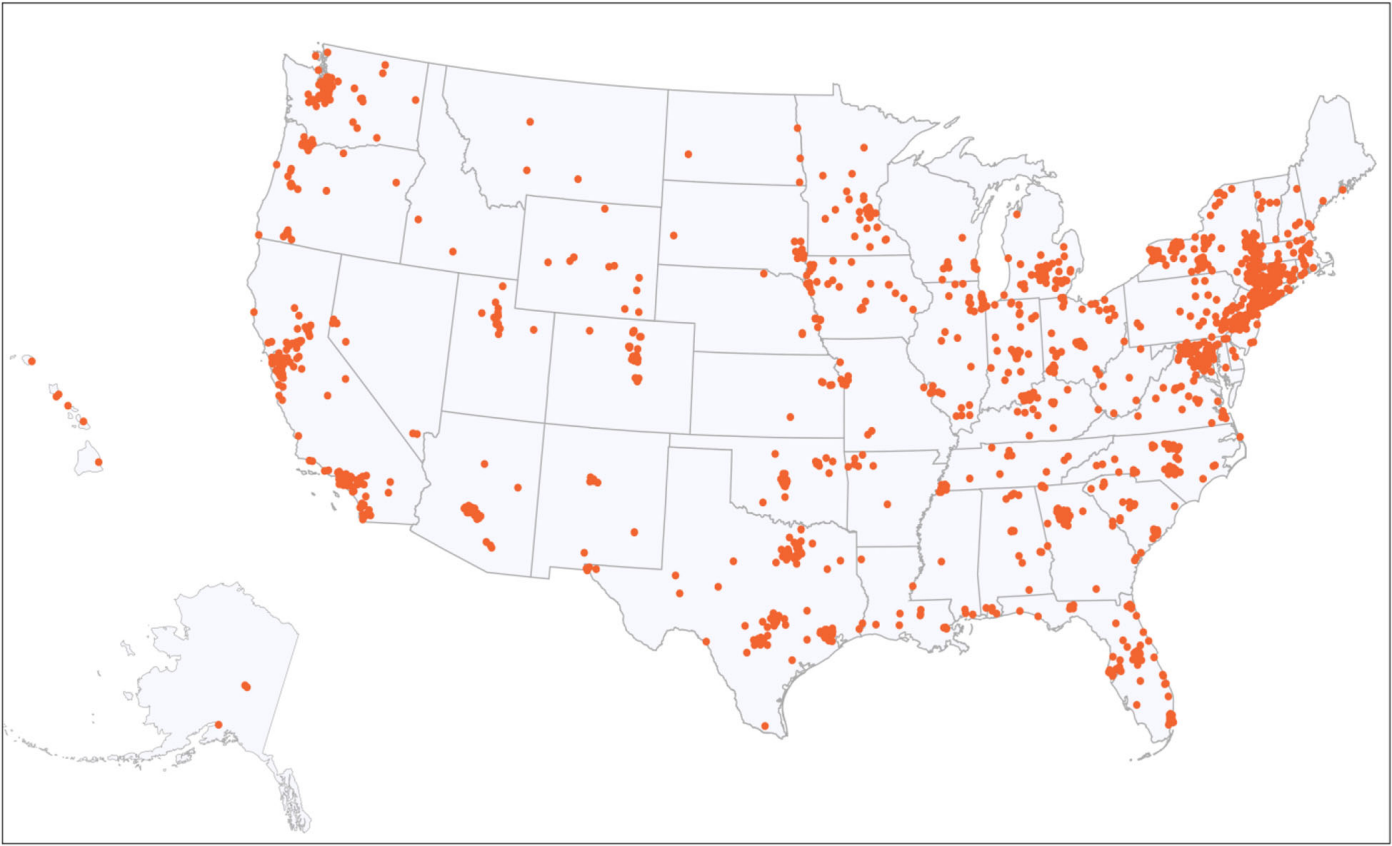
Map of zip codes, representing maternal residence at time of pregnancy

The majority of participants (64.5%) were between the ages of 25 and 35 when they gave birth; 13.5% were pregnant at the time of data collection. Most were born in the US (90%) and the majority completed post-secondary education. Participants from all 50 states completed the survey (see Fig. 2), and as expected, the largest proportion of responses were submitted by women from New York State (29.7%). One in three women across the whole sample reported family incomes less than $50,000 per year. The majority of participants received prenatal care from midwives (71.1%), and half (49.6%) gave birth in their homes or a freestanding birth center. Fewer women of colour had prenatal care by midwives (eg. 59.9%) compared to white women (76. 5%), and fewer women of colour (38.2%) compared to white women (55.2%) gave birth in homes or birth centers. Close to 14% of women had a Cesarean birth (CB), with variation by race: 17.8% women of colour had a CB compared to 11.8% of White women. Additional file 1: Table S2 displays socio-demographic characteristics for the 2700 participants, the 2138 participants included in the analysis of mistreatment items. Sample characteristics for the 2138 women included in the mistreatment analysis closely resembled those of all women who started the survey (*n* = 2700).

### How common is mistreatment?

One in six women (17.3%) in our sample experienced one or more types of mistreatment (Table 2). Being shouted at or scolded by a health care provider was the most commonly reported type of mistreatment (8.5%), followed by “health care providers ignoring women, refusing their request for help, or failing to respond to requests for help in a reasonable amount of time” (7.8%). Fewer women reported violation of physical privacy (5.5%), and health care providers threatening to withhold treatment or forcing them to accept treatment they did not want (4.5%). Very few women reported physical abuse, sharing of their personal information without consent, or healthcare providers threatening them in other ways (see Table 2). See Table 3 for quotes from the GVtM survey, illustrating mistreatment of US women.

**Table 2:** Mistreatment by Care Providers in Childbirth (MCPC) Indicators (*n* = 2138)

Did you experience any of the following issues or behaviours during your care?	*n* (%)
Your private or personal information was shared without your consent	26 (1.2)
Your physical privacy was violated (i.e., being uncovered or having people in the delivery room without your consent)	117 (5.5)
Health care providers (doctors, midwives, or nurses) shouted at or scolded you	182 (8.5)
Health care providers threatened to withhold treatment or to force you to accept treatment you did not want	97 (4.5)
Health care providers threatened you in any other way	44 (2.1)
Health care providers ignored you, refused your request for help, or failed to respond to requests for help in a reasonable amount of time	166 (7.8)
You experienced physical abuse (including aggressive physical contact, inappropriate sexual conduct, refusal to provide anesthesia for an episiotomy, etc.)	27 (1.3)
Any mistreatment (one or more of the above)	369 (17.3)

**Table 3:** Quotes illustrating mistreatment of US women

*Before I switched to a birth center, one military midwife was disrespectful of our cultural needs and refused to accept them. When I mentioned my desires, I was belittled and made to feel incompetent.* Hispanic woman who gave birth in California	
*The doctor who refused to test me for an amniotic fluid leak and instead tested me for an STD test I had already received during the pregnancy. I believe his assumption that I was leaking something due to an STD rather than a pregnancy complication was due to race and put my life and my newborns life at risk - I went a week leaking fluid after I had went in to get it checked out. I worry that Doctor is still discriminating against other mothers and they are receiving negligent care as well.* Black woman who gave birth in California	
*I was told I was hurting my children and being selfish because I wanted to have a vaginal delivery. Both children were in head down birth position. I was forced into a cesarean by my OB.* Indigenous woman who gave birth in Texas	
*The doctor who performed my c-section was hateful, rude, rough and threatening.* Indigenous woman who gave birth in Oklahoma	
*[I was] forced to be in a hospital because of having Medicaid which led to many interventions and being bullied/talked down to until I agreed. This pregnancy we saved up for a midwife so I can have a home birth.* Indigenous woman who gave birth in New York State	
*The amount of times I felt coerced into decisions or was mocked or rushed. Overall it was a very dehumanizing and frustrating experience … ..my original ob/gyn practice was rude and insulting to me and said that I risked having child protective services being called if I refused antibiotics due to being GBS positive.* White woman who gave birth in NJ	
*The forced episiotomy. The doctor didn’t care, refused to give me medication because my episiotomy hurt, Nurse XX from XX told me to get over it and gave me lube & told me to do anal sex instead! That’s the care we’re getting in Southern California if you are not insured & have to rely on Medical insurance.* Hispanic woman who gave birth in California	
When I refused to be induced-even after I was a couple days “overdue” I seriously started to feel like *I* was the problem. It was horrible. White woman who gave birth in Iowa at 24	
*I hated being shouted at and lied to by the midwife.. I never dreamed that a woman would treat a laboring woman that way. She was abusive and downright mean. I was refused food and water for 26 hours. I wasn’t allowed to move out of bed to walk around. I felt like I lost my autonomy over my own body. I had given up and I remember weeping when my son was born. I was at least glad he was safe. I felt like a child and I felt so unlike my usual self. These professionals broke my spirit.* Hispanic woman who gave birth at a in hospital birth center inside a hospital in North Carolina	
*The way I was treated during postpartum. If I was given adequate support with breastfeeding and actual education about it, I feel I would have been successful outright instead of struggling for months, and if I was not judged for being a younger mom, I would have felt safe and secure* South Asian woman who gave birth in Nevada	
*One nurse, whom we otherwise really liked, made comments generalizing about people by race (*e.g.*, “you Asian women all tear during birth”). It wasn’t done in a judgmental way but I would have preferred that she not say such things.* East Asian woman	
*I was offered WIC repeatedly though I explained that I did not qualify. I believe it was because I am Latina and my partner black that we were repeatedly offered WIC.* Hispanic woman with Black partner in New York	

### Mistreatment by sociodemographic factors

#### Race, ethnicity and immigration status

Indigenous women were the most likely to report experiencing at least one form of mistreatment by healthcare providers (32.8%), followed by Hispanic (25.0%) and Black women (22.5%). Women who identified as White were least likely to report that they experienced any of the mistreatment indicators (14.1%). Differences in mistreatment by race were pronounced for some indicators. For example, twice as many Hispanic and Indigenous women as compared to White women reported that health care providers shouted at or scolded them. Likewise, Black women, Hispanic women, Asian, and Indigenous women were twice as likely as White women to report that a health care provider ignored them, refused their request for help, or failed to respond to requests for help in a reasonable amount of time (see Table 4).

**Table 4:** Mistreatment indicators, stratified by maternal race (n = 2138)

	Black *n* = 320	Hispanic *n* = 188	Indigenous *n* = 64	Asian *n* = 90	Women of colour *n* = 682	White *n* = 1416
	*n* (%)	*n* (%)	*n* (%)	*n* (%)	*n* (%)	n (%)
Your private or personal information was shared without your consent	2 (0.6)	5 (2.7)	2 (3.1)	0 (0)	9 (1.3)	17 (1.2)
Your physical privacy was violated (i.e., being uncovered or having people in the delivery room without your consent)	27 (8.4)	12 (6.4)	6 (9.4)	7 (7.8)	52 (7.6)	62 (4.4)
Health care providers (doctors, midwives, or nurses) shouted at or scolded you	35 (10.9)	30 (16.0)	10 (15.6)	9 (10.0)	87 (12.8)	90 (6.4)
HCPs threatened to withhold treatment or to force you to accept treatment you did not want	21 (6.6)	11 (5.9)	7 (10.9)	6 (6.7)	45 (6.6)	51 (3.6)
Health care providers threatened you in any other way	6 (1.9)	8 (4.3)	3 (4.7)	1 (1.1)	18 (2.6)	26 (1.8)
Health care providers ignored you, refused your request for help, or failed to respond to requests for help in a reasonable amount of time	41 (12.8)	23 (12.2)	7 (10.9)	12 (13.3)	85 (12.5)	79 (5.6)
You experienced physical abuse (including aggressive physical contact, inappropriate sexual conduct, a refusal to provide anesthesia for an episiotomy, etc.)	6 (1.9)	4 (2.1)	0 (0)	1 (1.1)	11 (1.6)	16 (1.1)
Any mistreatment (one or more of the above)	72 (22.5)	47 (25.0)	21 (32.8)	19 (21.1)	162 (23.8)	199 (14.1)

Overall, White women with a White partner reported the least mistreatment (12.0%), followed by White women with a Black partner (17.0%) (see Additional file 1: Table S3). Bi-racial couples experienced less mistreatment when the woman was White as opposed to Black. However, for some indicators of mistreatment (eg., *Health care providers ignored you, refused your request for help, or failed to respond to requests for help in a reasonable amount of time*) White women with a Black partner were twice as likely to report mistreatment when compared to White women with a White partner.

Women who were born in the US reported similar rates of mistreatment compared to women who were not born in the US, but had lived there for more than 5 years (see Additional file 1: Table S4). Recent immigrants were more likely to report mistreatment, although results should be interpreted with caution as the number of recent immigrants was small (*n* = 34).

#### Age and parity

One in four women 24 or younger reported any mistreatment compared to one in seven women over 30 years old. Young women were also more likely to report physical abuse by providers compared to older women (Additional file 1: Table S5). Multiparous women reported lower rates of mistreatment on all indicators (see Additional file 1: Table S6), compared with women who were first-time mothers. Overall, first-time mothers were twice as likely to report mistreatment.

#### Socioeconomic, social, and pregnancy risk status

Women who reported low SES had similar rates of mistreatment on some of the indicators (e.g. sharing of personal information without consent) but were twice as likely to report being threatened or shouted at by HCPs, compared to women with moderate or high SES (Table 5). Women with pregnancy complications and women with social risks (i.e. a history of substance use, incarceration, and/or IPV) reported among the highest overall mistreatment rates among the subpopulations studied, with one in three reporting any mistreatment. These two groups were also more likely to report being shouted at or scolded and that their physical privacy was violated (Table 5).

**Table 5:** Mistreatment, stratified by SES, and elevated pregnancy/social risk (*n* = 2138)

	Low SES		Elevated pregnancy risks		Elevated social risks	
	Yes (*n* = 743)	No (*n* = 1395)	Yes (*n* = 441)^a^	No (*n* = 1697)	Yes (*n* = 176)^b^	No (*n* = 1962)
	*n* (%)	*n* (%)	*n* (%)	*n* (%)	*n* (%)	*n* (%)
Your private or personal information was shared without your consent	12 (1.6)	14 (1.0)	10 (2.3)	16 (0.9)	5 (2.8)	21 (1.1)
Your physical privacy was violated (i.e., being uncovered or having people in the delivery room without your consent)	47 (6.3)	70 (5.0)	37 (8.4)	80 (4.7)	23 (13.1)	94 (4.8)
Health care providers (doctors, midwives, or nurses) shouted at or scolded you	89 (12.0)	93 (6.7)	68 (15.5)	114 (6.7)	27 (15.3)	155 (7.9)
Health care providers threatened to withhold treatment or to force you to accept treatment you did not want	48 (6.5)	49 (3.5)	34 (7.7)	63 (3.7)	17 (9.7)	80 (4.1)
Health care providers threatened you in any other way	19 (2.6)	25 (1.8)	13 (2.9)	31 (1.8)	5 (2.8)	39 (2.0)
Health care providers ignored you, refused your request for help, or failed to respond to requests for help in a reasonable amount of time	78 (10.5)	88 (6.3)	53 (12.0)	113 (6.7)	23 (13.1)	143 (7.3)
You experienced physical abuse (including aggressive physical contact, inappropriate sexual conduct, a refusal to provide anesthesia for an episiotomy, etc.)	19 (2.6)	8 (0.6)	10 (2.3)	17 (1.0)	6 (3.4)	21 (1.1)
Any mistreatment (one or more of the above)	160 (21.5)	209 (15.0)	123 (27.9)	246 (14.5)	53 (30.1)	316 (16.1)

^a^Elevated pregnancy risk status: Women were grouped as having pregnancy risk factors if they reported a pre-pregnancy BMI of 40 or higher, were carrying twins, or reported that they experienced high blood pressure, gestational diabetes or other health complications during pregnancy (including breech baby, problems with baby’s growth/health, preterm labour, but not preterm birth)

^b^History of social risks: To distinguish those who may experience differential treatment because of social factors, we grouped together women who reported substance use (smoking or daily alcohol use during pregnancy, and/or drug dependence during pregnancy), women with a history of incarceration (herself or partner), involvement of child or family services, and/or reported intimate partner violence

### Mistreatment by context of care

#### Place of birth

Table 6 shows higher rates of mistreatment in hospital settings (28.1%), including birth centers that are located inside hospitals (24.0%), than in community birth settings (home or freestanding birth center). Rates of mistreatment were similar between women who gave birth at home (5.1%) or in a freestanding birth center (7.0%). The likelihood of being ignored by care providers and/or providers refusing to help was three times more common among women who gave birth in hospital settings (12.6 and 10.8%), compared to those who delivered at home (2.3%) or in a freestanding birth center (2.5%). Violation of physical privacy was also three times more common in hospital settings. Being threatened by care providers or having treatment withheld/being forced to accept treatment was twice as likely in hospital settings, compared to community settings.

**Table 6:** Mistreatment, stratified by actual place of birth (*n* = 1954)

	Hospital (*n* = 759)	Birth Centre Inside Hospital (*n* = 167)	Birth Centre Outside Hospital (*n* = 157)	Home (*n* = 871)	Transferred to hospital from community (*n* = 107)
	*n* (%)	*n* (%)	*n* (%)	*n* (%)	*n* (%)
Your private or personal information was shared without your consent	9 (1.2)	5 (3.0)	1 (0.6)	7 (0.8)	0 (0)
Your physical privacy was violated (i.e., being uncovered or having people in the delivery room without your consent)	78 (10.3)	15 (9.0)	1 (0.6)	7 (0.8)	13 (12.1)
Health care providers (doctors, midwives, or nurses) shouted at or scolded you	98 (12.9)	18 (10.8)	4 (2.5)	19 (2.2)	28 (26.2)
Health care providers threatened to withhold treatment or to force you to accept treatment you did not want	50 (6.6)	7 (4.2)	5 (3.2)	16 (1.8)	10 (9.3)
Health care providers threatened you in any other way	19 (2.5)	4 (2.4)	4 (2.5)	6 (0.7)	9 (8.4)
Health care providers ignored you, refused your request for help, or failed to respond to requests for help in a reasonable amount of time	96 (12.6)	18 (10.8)	4 (2.5)	20 (2.3)	19 (17.8)
You experienced physical abuse (including aggressive physical contact, inappropriate sexual conduct, a refusal to provide anesthesia for an episiotomy, etc.)	16 (2.1)	3 (1.8)	1 (0.6)	1 (0.1)	4 (3.7)
Any mistreatment (one or more of the above)	213 (28.1)	40 (24.0)	11 (7.0)	44 (5.1)	37 (34.6)

Women who were transferred from a community setting to a hospital, after the onset of labor, experienced high rates of mistreatment (34.6%). One in four reported being shouted at or scolded by a health care provider, one in ten were threatened, and one in seven were ignored (Table 6). Of the women who transferred to hospital from a home birth (*n* = 80), 37 (46.3%) reported that they were treated poorly by health professionals during their transfer or afterwards because of their decision to have a home birth.

#### Mode of delivery

Additional file 1: Table S7 shows much higher rates of mistreatment when women had unplanned Cesareans and instrumental vaginal births. Women who had a vaginal birth after caesarean (VBAC) reported low levels of mistreatment. Separating women who had a VBAC in a community birth setting versus in a hospital revealed that 1 in 3 women who had a VBAC in the hospital experienced mistreatment versus 6% of women who gave birth in the community.

#### Newborn health problems

One in four women who reported that their newborn(s) had any health problems experienced one or more types of mistreatment. Women whose newborns had health problems were more likely to report that their private or personal information was shared without their consent and that providers ignored them, refused their request for help, or failed to respond to requests for help in a reasonable amount of time, compared to women whose newborns did not have health problems (see Additional file 1: Table S8).

#### Disarticulation between provider and woman

We found higher rates of mistreatment when preferences for care did not align between women and providers: Any mistreatment was reported by 19.4% of women who declined care during pregnancy or birth, 37.9% of women who reported being pressured into one or more medical interventions or procedures, and 78.8% if they also had a difference in opinion with their care provider (see Additional file 1: Table S9).

### Demographic and other factors related to mistreatment

In bivariable logistic regression analyses (Table 7), we found that Black, Hispanic and Indigenous women, primiparas and women with elevated pregnancy risks were significantly more likely to report mistreatment, compared with White women. Younger women, women with a history of substance use, incarceration and/or interpersonal violence (IPV) and those of low socio-economic status also reported significantly increased odds of mistreatment compared with those that did not have these sociodemographic risk factors for mistreatment (see Table 7). Finally, context of care was linked to mistreatment. Women who had prenatal care from midwives were much less likely to report mistreatment compared to those who had prenatal care from physicians (OR 0.31, 95% CI 0.25–0.40), whereas an unplanned Cesarean or assisted vaginal birth was linked to significantly increased odds of mistreatment compared to spontaneous vaginal delivery (OR 3.7, 95% CI 2.8–5.0). Women who gave birth at the hospital were 7 times as likely to report any mistreatment compared to women who gave birth in the community (OR 7.2, 95% CI 5.3–9.7). Women who reported a difference in opinion with their care provider had very high odds of mistreatment compared with those who did not report a difference in opinion (OR 22.7, 95% CI 13.9–36.9).

**Table 7:** Crude odds ratios estimating associations between maternal characteristics and any mistreatment (*n* = 2138)

	n	OR	95% CI	
		Logistic Regression	Lower bound	Upper bound
SOCIO-DEMOGRAPHICS				
Maternal Race: Black (reference category: white)	2098	1.77	1.31	2.40
Maternal Race: Hispanic (reference category: white)	2098	2.04	1.42	2.93
Maternal Race: Asian (reference category: white)	2098	1.64	0.97	2.77
Maternal Race: Indigenous (reference category: white)	2098	2.98	1.73	5.13
Maternal Race: Women of colour (reference category: white women)	2098	1.91	1.51	2.41
Age: 17 to 25 years (reference category: 31–39)	1956	1.71	1.08	2.69
Age: 26–30 years (reference category: 31–39)	1956	1.15	0.88	1.49
Age: Over 40 (reference category: 31–39)	1956	1.04	0.62	1.74
Nulli/primiparity (reference category: multiparity)	2135	2.50	1.99	3.14
Low SES - Yes (reference category: no)	2138	1.56	1.24	1.96
MEDICAL OR SOCIAL FACTORS				
Elevated pregnancy risk - Yes (reference category: no)	2138	2.28	1.78	2.92
History of substance use, incarceration and/or IPV (social risk)- Yes (reference category: no)	2138	2.24	1.59	3.17
CONTEXT OF CARE				
Prenatal midwifery care (reference group: prenatal physician care)	2076	0.31	0.25	0.40
Actual place of birth hospital or alongside birthing center (reference group: community birth)	2119	7.17	5.31	9.68
Mode of birth unplanned Cesarean or operative vaginal delivery (reference group: planned Cesarean or spontaneous vaginal birth)	2129	3.72	2.79	4.97
Difference in opinion with care provider (reference group: no difference in opinion with care provider)	2138	22.69	13.94	36.92

### Intersection between race, other maternal characteristics, and context of care

When examining the intersection of race and the maternal characteristics, rates of mistreatment among women of colour who were young, nulliparous or primiparous, or had low SES, social risk factors, or pregnancy complications were higher than for white women who reported the same conditions or experiences. For example, among those who had pregnancy complications, mistreatment was reported by 37.0% women of colour versus 22.1% white women. Similarly, women of colour with low SES reported higher rates of mistreatment than white women with low SES (26.9% versus 17.7%). Regardless of race, among women who had a difference in opinion with their care provider, the majority (83.0% of women of colour, 76.4% of white women) reported one or more types of mistreatment (Table 8).

**Table 8:** Intersection between mistreatment, race and additional variables (n = 2138)

		n (%) who report any mistreatment	
Intersectional Factor	n	Women of colour (*n* = 162)	White women (*n* = 199)
Sociodemographics			
Nulliparity	811	92/282 (32.6)	114/529 (21.6)
Age 17–25 years	116	17/55 (30.9)	11/61 (18.0)
Low SES	726	83/309 (26.9)	74/417 (17.7)
Medical or Social Factors			
Elevated pregnancy risk	434	60/162 (37.0)	60/272 (22.1)
Social risk	172	30/66 (45.5)	21/106 (19.8)
Context of care			
Prenatal midwifery care	1120	63/393 (16.0)	107/1057 (10.1)
Actual place of birth: hospital or in-hospital birthing centre	1013	137/404 (33.9)	146/609 (24.0)
Actual place of birth: home or freestanding birthing centre	1009	17/258 (6.6)	38/751 (5.1)
Unplanned Caesarean or operative vaginal birth	235	43/105 (41.0)	48/130 (36.9)
Difference in opinion with care provider	102	39/47 (83.0)	42/55 (76.4)

Place of birth and operative birth appear to have similar modification effects for both women of color and white women. Giving birth at home or in a freestanding birth center was associated with lower rates of mistreatment across racial groups, when compared to rates of mistreatment among women who gave birth in hospitals. For example, among women of colour who gave birth in the community, 6.6% reported any mistreatment, compared to 33.9% who gave birth at hospitals.

## Discussion

In the Giving Voice to Mothers study, service users of maternity care in the US described mistreatment across categories that closely align with the WHO (Bohren) typology that was derived from global evidence on the phenomena. In this study of care in a high resource country, physical abuse was uncommon, but verbal abuse and failure to respond to requests for help were the most common types of reported mistreatment; rights to information and autonomy were apparently disregarded; and difference of opinion with care providers had a strong association with reported mistreatment. While the overall rates of mistreatment are lower in our US sample than recent studies report in low resource settings [5], they are still unacceptably high for a high resource country given a cultural emphasis on autonomy, gender equity, human rights, better working conditions for providers, and resources for training.

Protective factors, in terms of mistreatment were: being White, having a vaginal birth, giving birth at home or in a freestanding birth center, having a midwife as the primary prenatal provider, and having a baby after 30 years of age. Being multiparous was also protective, which may suggest that prior experience helps patients avoid disrespectful treatment, or conversely that disrespectful treatment is normalized by prior experiences among certain populations. Importantly, more than half of our sample planned community births, and they experienced very low rates of mistreatment when compared to those who gave birth in hospital. Since less than 2% of all childbearing women in the US give birth in community settings [41], the rate of mistreatment (30%) among women in our sample who gave birth in a hospital, is likely a better estimate of the true rate of mistreatment during childbirth among US women.

### Patient-led measurement of health equity

In 2017 the National Quality Forum (NQF) convened a multi-stakeholder group of experts to develop a shared agenda to achieve health equity [42]. The team highlighted four priority areas for action: identify and prioritize areas to reduce health disparities, invest in the development and application of person-centered health equity performance measures, incentivize the reduction of health disparities, and implement evidence-based interventions to reduce disparities.

Our Giving Voice to Mothers study has addressed this mandate through the patient-led development and validation of unique items that can be used to measure disrespect, abuse, and discrimination during maternity care. Using these items, we were able to show that some populations experienced significantly higher rates of mistreatment, such as women of color, young women, and those who reported economic, social or health risks. All women who self-identified as Black, Indigenous, Hispanic, or Asian reported higher than average experiences of mistreatment. Regardless of their own race, having a partner who was Black also increased their risk of mistreatment.

The types and recipients of mistreatment identified by participants in the GVtM study are consistent with patient-oriented research evidence from a recent qualitative study [43] in California. McLemore and colleagues [43] explored pregnancy-related healthcare experiences through focus groups of women of color from three urban areas in California. The study included English and Spanish speaking women, age 18 or greater with social and/or medical risk factors for preterm birth. Based on the data collected from 54 women in two focus groups, the authors identified five themes: 1) disrespect during healthcare encounters; 2) stressful interactions with all levels of staff; 3) unmet information needs; 4) inconsistent social support; and 5) care that affected confidence in parenting and newborn care. Focus group participants provided examples of each of the seven types of mistreatment that we measured. Participants discussed sharing of personal information, violation of physical privacy and being “yelled at” by a physician. Half of the participants discussed being pressured or threatened, with the most common type of threat being, “if you do not comply or do this, your baby will die or you will have a bad outcome.” Similarly, coercive language reported by participants in our GVtM study frequently referred to the potential loss of the baby.

### Mistreatment, inequity, and access to high quality care

In high resource countries, pregnant people who experience discrimination due to lower socioeconomic status, race/ethnicity, or housing instability, are especially at risk for poor health outcomes [20]. For, example, a European team reviewed published evidence on discrimination against Romani women in maternity care in Europe [21]. Results revealed that many Romani women encounter barriers to accessing maternity care. Even when they were able to access care, they experienced discriminatory mistreatment on the basis of their ethnicity, economic status, place of residence or language. The grey literature revealed some health professionals held underlying negative beliefs about Romani women [21].

Similarly, much has been written about how implicit bias by healthcare provider links to disparities in access to and quality of care [44]. Growing evidence suggests that differential quality of care in North America contributes to racial and ethnic disparities in obstetric and perinatal outcomes [18, 20, 45–47] and that access to high quality of care in obstetrics varies widely by jurisdiction and type of provider [48]. In our study Indigenous women were the most likely to report mistreatment among the racial groups, closely followed by African American and Hispanic women. Indigenous men and women in Central America report barriers to accessing healthcare and abusive treatment and neglect of professional ethics from HCPs [49]. Canadian research has documented the distress and racism experienced by Aboriginal women including discrimination, loss of autonomy and dehumanizing interactions with care providers [50].

Vedam et al. [32] found that in British Columbia, women from vulnerable populations (i.e. recent immigrants or refugees, women with a history of incarceration and/or substance use, homelessness or poverty), women with pregnancy complications, those who have birth at hospital (versus home) and women who experienced pressure to have interventions were more likely to score very low on the MOR index, a scale that measures respectful maternity care [32]. Our intersectional analysis underscores that the negative impacts of race and social vulnerability are intertwined and cumulative, that those who are already at risk for the worst outcomes, also experience higher levels of mistreatment. Given that the burden of disparities borne by these populations has shown little improvement in recent decades, understanding the presence of mistreatment in childbirth may aid our efforts to comprehend underlying causes, and inform our efforts to eliminate them.

### The context of care

We also elicited differential treatment when women’s choices and opinions about “the right care” for themselves or their baby did not align with providers. Those who were transferred to hospital from the community, women who reported being pressured into interventions, and those who had a difference of opinion with their health care provider reported higher rates of mistreatment. Differential rates of mistreatment may be associated with differences by race in level of patient autonomy and/or pressure to accept interventions from providers, which in itself constitutes mistreatment. The relationships between differences of opinion, interventions, and mistreatment require further study to elucidate the temporal nature of these associations. In qualitative study, researchers in New England interviewed 50 white women and 32 women of color the day after they gave birth at a tertiary care facility [51]. Women of color reported more pressure to accept epidural anesthesia and were also more likely to experience failure in their pain medication and report that providers ignored their pain and anxiety.

Higher rates of mistreatment among those who have unplanned cesarean births warrants a closer examination, given country-level disparities in overuse and underuse of obstetric interventions [1], as well as the confounding reality that proportionately more women of colour in our sample, as in the general US population, had cesareans. Multiple authors have examined racial differences in both primary cesarean and VBAC rates and found women of colour have an increased risk of cesarean delivery after adjusting for sociodemographic and clinical risk factors [52–55]. Additionally, women with private health insurance have a lower predicted probability of having a cesarean section for clinical indications than do women with public health insurance [56].

The significant number of respondents that reported “being ignored” or that “providers failed to respond to their requests for help” is a disturbing finding in a high resource setting, especially in light of recent data that links delayed response to clinical signs to maternal mortality. The California Department of Public Health (CDPH), the California Maternal Quality Care Collaborative (CMQCC) and the Public Health Institute (PHI) recently released data from a statewide examination of maternal deaths from 2002 to 2007 [57]. The report identified that healthcare provider factors were the most common type of contributor to maternal deaths, averaging 2.5 factors per case and present among 269 cases or 81% of maternal deaths in that time period. The most common provider factor was delayed response to clinical warning signs, followed by ineffective care [57].

Finally, place of birth appears to have a modulating effect on experiences of mistreatment. Women from all race and ethnic backgrounds who gave birth at home or in birth centers reported far fewer examples of all seven types of disrespect and abuse. This is especially poignant in light of the finding that women who needed to transfer to hospital from a planned community birth, ostensibly to access a safe environment to respond to emerging complications, experienced very high rates of mistreatment. Whether these differences are a result of the change in locus of control and loss of cultural safety that all people feel in their own environments [58], or the effects of structural racism, societal norms, and implicit bias that exist in institutional cultures, remains to be explored.

### Implications

Bohren and colleagues argue that instances of mistreatment constitute violations of people’s human rights. [13] Several respondents in our study provided descriptions about how mistreatment violated these basic principles. Amnesty International identified the inappropriate, disrespectful, and discriminatory treatment of pregnant and childbearing people in the United States as constituting a human rights violation and documented incidents of women, particularly women of colour, being abandoned, ignored, threatened, coerced, shouted at, and otherwise mistreated [59]. Violations of human rights in childbirth tend to be more severe in countries where women have limited options in terms of where, how and with whom they can give birth. Authors of the WHO Research Group [60] argue that, to prevent mistreatment, health care providers need to first consider how they can meet women’s socio-cultural, emotional and psychological needs.

A recent publication on addressing racial disparities in the management of hypertension discussed how performance measures can be used to incentivize self-monitoring programs, and the development of pragmatic, effective interventions to improve health equity [61]. The authors describe a multi-strategy approach that takes into account the complex interactions between social determinants of health, societal drivers of inequity, payment models, and cultural competency education for health professionals. They refer to the five domains of health equity measurement described in the NQF report: first, building collaborations to address factors that maintain racial and ethnic disparities; second, creating a culture of equity and individualized care and routine training around issues of structural racism and intersectionality of multiple drivers of disadvantage; third, moving to the development of multidisciplinary teams, and fourth, addressing issues of access to high quality care across communities and settings for care. The final domain focusses on the equitable application of evidence-based interventions that are responsive to patient reported outcomes and priorities [61].

With respect to mistreatment, dignity, and freedom from human rights abuses in maternity care, this last priority is dependent on the health systems ability to monitor and describe patient experience with reliable indicators. Our patient-driven performance measures can target the key components of mistreatment to address by jurisdiction, and identify settings where quality improvement related to respectful maternity care is most needed, as well service users most at risk for differential treatment. Abuya and colleagues [19] have suggested several intervention and implementation activities to eliminate mistreatment of women in low resource countries. Many of these strategies are also relevant in the US context, such as training for care providers in promoting respectful care including values clarification and attitude transformation (VCAT), training on VCAT based on providers’ and clients’ rights and obligations, and revision of professional ethics and practices. The authors also recommend strengthening facility quality improvement systems for monitoring, reporting, addressing, and resolving disrespect and abuse cases. Mentorship and on-the-job role-modeling by identified champions within the facility as part of routine continuous professional education has been shown to shift team culture. At the same time civic education about patient rights and avenues for redress may be needed to ensure accountability even in high resource countries.

## Strengths and limitations

Strengths of the study include the large sample size that allows for the best estimate to date of the frequencies and types of mistreatment occurring among diverse subpopulations among childbearing people in the US. Importantly, the Giving Voice to Mothers study provides the first complete set of patient-designed and validated quantitative indicators, across all domains of the Bohren typology, that can be used to describe prevalence and characteristics of mistreatment in maternity care across all settings. This study also provides the first published estimates of associations between social factors like race/ethnicity, and modulating effects of planned place of birth or interventions, and rates and forms of mistreatment as identified by patients themselves.

A primary limitation of the study is that the sample is voluntary and not population-based, as there is currently no data collection system designed to capture and describe experiences of birth care for all pregnant people in the United States. Rather we sampled for diversity, oversampling from communities that are often under-represented in national studies on experience of care, such as Black and Indigenous women, and those planning to give birth at home or in a birth center. Compared to the characteristics of women who gave birth in the United states in 2016, women in our study had similar proportions of previous births, but were more educated, older, and more likely to have been born in the United States [62]. With respect to racial representativeness, we report data from a similar proportion of black women and more Indigenous women; 14.0% of US births in 2016 (CDC) were women who identified as ‘black’ compared to 15.4% in this study; 1% are identified as Indigenous in the US vs 3% in our sample [62]. Overall, our samples of women from Hispanic, Asian, and other communities of color were lower than the national reported rates. Of note, 24% of the US births in 2016 had a mother identified as “Hispanic origin” compared to roughly 10% in the current study.

Notably, patient reports of improved experience of care in homes and birth centers are repeatedly cited in the global literature. Since 50% of our sample were reporting on community births (when the representative rate would have been 2%), the logical expectation would that the entire sample is skewed towards much ***less*** mistreatment than the general population. Because women with very positive or very negative experiences are often more motivated to participate in studies that invite them to share their stories, we anticipate that we have lower representation from women who had more routine or simply “satisfactory” experiences that might not be characterized as either particularly empowering nor traumatizing. To mitigate bias introduced because communities of color tended to describe worse experiences and community birthers more positive ones, we stratified results by race and place of birth.

In general, the GVTM sample might have a ‘higher’ SES population than is representative of the US childbearing population which, given our findings, we anticipate would decrease rates of reported mistreatment, and potentially underestimate mistreatment in the US population at large. The large proportion of community birth also accounts for the higher socioeconomic status – since without universal health care, community birth is often not accessible by low SES service users. Since even in this more privileged population the overall rates of mistreatment were at 17%, and significantly higher for those who planned and delivered in hospitals, our findings highlight the need for further investigations in this understudied area.

Regional variation in outcomes and access to high quality care across the United States have been described in the literature [48], and our national sample is not representative of the lived experience of many subgroups including undocumented immigrants, incarcerated pregnant parents, and families located in rural settings with limited options for maternity care. With respect to generalizability in the international context, women and people have different interpretations of consent and power. Hence, while standardizing indicators through these typologies is helpful, it will not change that each person will have their own sense of bodily/self autonomy and human rights, placed within the cultural context of each environment. Finally, not all people giving birth identify as women and/or mothers, and mistreatment as associated with gender identity, sexuality and parenting status are areas where further study is needed.

Nonetheless, that higher rates of mistreatment so clearly track along marginalized groups, and with women whose choices in care differ from their providers’ recommendations, suggests that regardless of any sampling issues invariably contained in this study, there is much work yet to be done in the United States, as no level of mistreatment of a childbearing person is acceptable.

## Conclusion

The Giving Voice to Mothers- US study led to development of several new patient-designed indicators of mistreatment in maternity care. They use lay language to capture lived experience from the service user’s perspective, and can be used to quantify the nature and frequency of occurrence of different types of disrespect and abuse. They are aligned closely with global definitions of the domains of mistreatment, and thus are relevant across high, middle, and low resource countries.

Application of these measures elicited disparities in experience of maternity care across communities of color and birth settings in the United States. With some translation and adaptation, these indicators could be implemented in patient-reported outcomes research globally. In the United States, these indicators could be incorporated as performance measures to incentivize expansion of programs to address settings, practices, and institutional cultures that lead to persistent disparities in maternity care.

## Additional file

Additional file 1: **Table S1.** Self-identified maternal and paternal race (*n* = 2700). **Table S2.** Socio-demographic characteristics of samples, compared to national statistics. **Table S3.** Mistreatment, stratified by self-identified race of woman and partner. **Table S4.** Mistreatment, stratified by immigration status. **Table S5.** Mistreatment, stratified by maternal age at birth. **Table S6** Mistreatment, stratified by parity. **Table S7.** Mistreatment, stratified by labour induction and mode of birth. **Table S8.** Mistreatment, stratified by newborn health problems. **Table S9.** Mistreatment indicators, stratified by disarticulation between women and providers.

## Abbreviations

CA-PAMR: California Pregnancy-Associated Mortality Review
CDPH: California Department of Public Health
CI: Confidence Interval
CMQCC: California Maternal Quality Care Collaborative
GVtM - US: Giving Voice to Mothers–United States
HCP: Healthcare provider
IPV: Intimate Partner Violence
MADM: Mother’s Autonomy in Decision-Making Scale
MORi: Mothers on Respect Index
NGO: Non-governmental organization
NQF: National Quality Forum
PHI: Public Health Institute
PR: Perceptions of Racism
RMC: Respectful maternity care
SES: Socio-economic status
VBAC: Vaginal Birth after Cesarean
WHO: World Health Organization
