# Supplementary material for: The Giving Voice to Mothers study: inequity and mistreatment during pregnancy and childbirth in the United States
Source: Reprod Health. 2019 Jun 11;16:77. doi: 10.1186/s12978-019-0729-2 (PMC6558766; doi:10.1186/s12978-019-0729-2)
Supplement: Supplementary file 1 — Table S1. Self-identified maternal and paternal race (n = 2700). Table S2. Socio-demographic characteristics of samples, compared to national statistics. Table S3. Mistreatment, stratified by self-identified race of woman and partner. Table S4. Mistreatment, stratified by immigration status. Table S5. Mistreatment, stratified by maternal age at birth. Table S6 Mistreatment, stratified by parity. Table S7. Mistreatment, stratified by labour induction and mode of birth. Table S8. Mistreatment, stratified by newborn health problems. Table S9. Mistreatment indicators, stratified by disarticulation between women and providers. [file 12978_2019_729_MOESM1_ESM.docx]

**Supplemental tables**

**Table S1. Self-identified maternal and paternal race (n=2700)**

|  | n (%) | |
| --- | --- | --- |
| Self-identified as: | Maternal race | Paternal race |
| 1. Black (identified as Black or African in any of the race fields) | 380 (14.1) | 338 (12.5) |
| 2. Indigenous (identified as Native American/Native Alaskan/Native Hawaiian/Indigenous to Mexico or South America in any of the race fields but not as Black or African) | 77 (2.9) | 43 (1.6) |
| 3. Asian (identified as Asian in any of the race fields but not as Black/African/Indigenous) | 117 (4.3) | 93 (3.4) |
| 4. Hispanic (identified as Hispanic in any of the race fields, but not as Black/African/Indigenous/Asian | 241 (8.9) | 225 (8.3) |
| 5. Other person of colour | 24 (0.9) | 52 (2.0) |
| 6.White (identified as White in any of the race fields, and were not already allocated to groups 1-5) | 1651 (61.1) | 1714 (63.5) |
| Missing cases | 210 (7.8) | 235 (8.7) |

**Table S2: Socio-demographic characteristics of samples, compared to national statistics**

|  |  | n=2700 | n=2138 | National statistics 2016* |
| --- | --- | --- | --- | --- |
| Characteristic |  | % | % | % |
| Number of previous pregnancies | 1  2  3  4 or more | 22.8  28.4  22.3  26.5 | 23.8  27.6  22.7  25.9 | -  -  -  - |
| Number of previous births | 0  1  2-3  4 or more | 1.2  37.7  49.7  11.4 | 0.1  38.4  49.9  11.6 | -  37.0  50.0  13.0 |
| Pregnant at time of data collection | Yes  No | 13.5  86.5 | 13.0  87.0 | -  - |
| Maternal age at birth | 18-19  20-24  25-29  30-35  35+ | 0.3  5.4  23.8  40.1  30.4 | 0.3  5.4  24.4  40.1  29.8 | 4.0  21.0  30.0  29.0  17.0 |
| Maternal race | Black  Indigenous  Asian  Hispanic  White  Other | 15.4  3.1  4.7  9.8  67.0  -- | 15.4  3.1  4.3  9.0  68.1  -- | 14.0  1.0  7.0  23.0  52.0  3.0 |
| Born in US | Yes  No | 89.6  10.4 | 90.0  10.0 | 76.5  23.3 |
| Highest level of education | High school  Some college  Associate degree  Bachelor’s degree  Graduate degree  Other | 3.3  16.9  7.8  29.7  29.8  12.5 | 3.1  17.0  7.9  29.7  29.4  12.9 | 25.2  20.7  8.3  20.2  11.8  -- |
| Main source of payment for maternity care | Medicaid/CHIP  Private insurance  Self- pay  Other | 13.5  50.8  19.0  16.7 | 14.0  54.2  20.6  11.2 | --  --  --  -- |
| Total household income before taxes | $ 0-19,999  $ 20,000-49,999  $ 50,000-99,999  $ 100,000-159,999  $ 160,000- over | 5.8  23.1  35.0  22.3  13.8 | 5.7  22.9  35.1  22.5  13.9 | --  --  --  --  -- |

*Singleton hospital birth to women 18+, United States 2016

United States Department of Health and Human Services (US DHHS), Centers for Disease Control and Prevention (CDC), National Center for Health Statistics (NCHS), Division of Vital Statistics, Natality public-use data 2007-2017, on CDC WONDER Online Database, October 2018. Accessed at http://wonder.cdc.gov/natality-current.html on Apr 14, 2019 7:39:28 AM

**Table S3**: **Mistreatment, stratified by self-identified race of woman and partner**

|  | Woman is white, partner is white  (n = 1403) | Woman is black, partner is black  (n = 260) | Woman is white, partner is black  (n = 47) | Woman is black, partner is white  (n = 78) |
| --- | --- | --- | --- | --- |
|  | n (%) | n (%) | n (%) | n (%) |
| Your private or personal information was shared without your consent | 12 (0.9) | 2 (0.8) | 0 (0) | 0 (0) |
| Your physical privacy was violated (i.e., being uncovered or having people in the delivery room without your consent) | 55 (3.9) | 16 (6.2) | 1 (2.1) | 7 (9.0) |
| HCPs (doctors, midwives, or nurses) shouted at or scolded you | 77 (5.5) | 27 (10.4) | 3 (6.4) | 10 (12.8) |
| Health care providers threatened to withhold treatment or to force you to accept treatment you did not want | 41 (2.9) | 13 (5.0) | 1 (2.1) | 6 (7.7) |
| Health care providers threatened you in any other way | 22 (1.4) | 2 (0.8) | 0 (0) | 3 (3.8) |
| Health care providers ignored you, refused your request for help, or failed to respond to requests for help in a reasonable amount of time | 68 (4.8) | 28 (10.8) | 4 (8.5) | 12 (15.4) |
| You experienced physical abuse (including aggressive physical contact, inappropriate sexual conduct, a refusal to provide anesthesia for an episiotomy, etc.) | 11 (0.8) | 2 (0.8) | 0 (0) | 1 (1.3) |
| Any mistreatment (one or more of the above) | 169 (12.0) | 51 (19.6) | 8 (17.0) | 19 (24.4) |

**Table S4**: **Mistreatment, stratified by immigration status**

|  | Born in USA  n=1839 | Not born in USA but lived in US > 5 years  n= 171 | Not born in USA and lived in US for 5 years or less  n=34 |
| --- | --- | --- | --- |
|  | n (%) | n (%) | n (%) |
| Your private or personal information was shared without your consent | 23 (1.3) | 0 (0) | 1 (2.9) |
| Your physical privacy was violated (i.e., being uncovered or having people in the delivery room without your consent) | 98 (5.3) | 12 (7.1) | 2 (5.9) |
| Health care providers (doctors, midwives, or nurses) shouted at or scolded you | 155 (8.4) | 14 (8.2) | 4 (11.8) |
| Health care providers threatened to withhold treatment or to force you to accept treatment you did not want | 77 (4.2) | 9 (5.3) | 3 (8.8) |
| Health care providers threatened you in any other way | 35 (1.9) | 3 (1.8) | 2 (5.9) |
| Health care providers ignored you, refused your request for help, or failed to respond to requests for help in a reasonable amount of time | 136 (7.4) | 17 (10.0) | 4 (11.8) |
| You experienced physical abuse (including aggressive physical contact, inappropriate sexual conduct, a refusal to provide anesthesia for an episiotomy, etc.) | 23 (1.3) | 2 (1.2) | 0 (0) |
| Any mistreatment (one or more of the above) | 310 (16.9) | 29 (17.1) | 8 (23.5) |

**Table S5: Mistreatment, stratified by maternal age at birth**

|  | 17-24  (n = 116) | 25-30  (n = 654) | 31-39  (n = 1132) | 40-48  (n = 117) |
| --- | --- | --- | --- | --- |
|  | n (%) | n (%) | n (%) | n (%) |
| Your private or personal information was shared without your consent | 0 (0) | 8 (1.2) | 13 (1.1) | 1 (0.9) |
| Your physical privacy was violated (i.e., being uncovered or having people in the delivery room without your consent) | 9 (7.8) | 38 (5.8) | 59 (5.2) | 7 (6.0) |
| Health care providers (doctors, midwives, or nurses) shouted at or scolded you | 14 (12.1) | 64 (9.8) | 84 (7.4) | 10 (8.5) |
| Health care providers threatened to withhold treatment or to force you to accept treatment you did not want | 4 (3.4) | 36 (5.5) | 45 (4.0) | 5 (4.3) |
| Health care providers threatened you in any other way | 3 (2.6) | 12 (1.8) | 24 (2.1) | 2 (1.7) |
| Health care providers ignored you, refused your request for help, or failed to respond to requests for help in a reasonable amount of time | 19 (16.4) | 62 (9.5) | 67 (5.9) | 11 (9.4) |
| You experienced physical abuse (including aggressive physical contact, inappropriate sexual conduct, a refusal to provide anesthesia for an episiotomy, etc.) | 5 (4.3) | 10 (1.5) | 10 (0.9) | 2 (1.7) |
| Any mistreatment (one or more of the above) | 28 (24.1) | 121 (18.5) | 178 (15.7) | 19 (16.2) |

**Table S6: Mistreatment, stratified by parity**

|  | Nulli or Primiparity  (n=821) | Multiparity  (n=1314) |
| --- | --- | --- |
|  | n (%) | n (%) |
| Your private or personal information was shared without your consent | 15 (1.8) | 11 (0.8) |
| Your physical privacy was violated (i.e., being uncovered or having people in the delivery room without your consent) | 69 (8.4) | 47 (3.6) |
| Health care providers (doctors, midwives, or nurses) shouted at or scolded you | 99 (12.1) | 82 (6.2) |
| Health care providers threatened to withhold treatment or to force you to accept treatment you did not want | 52 (6.3) | 44 (3.3) |
| Health care providers threatened you in any other way | 31 (3.8) | 13 (1.0) |
| Health care providers ignored you, refused your request for help, or failed to respond to requests for help in a reasonable amount of time | 92 (11.2) | 73 (5.6) |
| You experienced physical abuse (including aggressive physical contact, inappropriate sexual conduct, a refusal to provide anesthesia for an episiotomy, etc.) | 15 (1.8) | 12 (0.9) |
| Any mistreatment (one or more of the above) | 209 (25.5) | 158 (12.0) |

**Table S7: Mistreatment, stratified by labour induction and mode of birth**

|  | Labor induction^1^  n=318 | Planned CS  n=85 | Unplanned CS  n=209 | Vaginal birth  n=1802 | Instrumental vaginal birth  n=33 | VBAC^2^  n=152 |
| --- | --- | --- | --- | --- | --- | --- |
|  | n (%) | n (%) | n (%) | n (%) | n (%) | n (%) |
| Your private or personal information was shared without your consent | 8 (2.5) | 0 (0) | 3 (1.4) | 22 (1.2) | 1 (3.0) | 0 (0) |
| Your physical privacy was violated (i.e., being uncovered or having people in the delivery room without your consent) | 34 (10.7) | 4 (4.7) | 22 (10.5) | 86 (4.8) | 5 (15.2) | 9 (5.9) |
| Health care providers (doctors, midwives, or nurses) shouted at or scolded you | 54 (17.0) | 8 (9.4) | 44 (21.1) | 121 (6.7) | 9 (27.3) | 14 (9.2) |
| Health care providers threatened to withhold treatment or to force you to accept treatment you did not want | 27 (8.5) | 1 (1.2) | 23 (11.0) | 71 (3.9) | 2 (6.1) | 10 (6.6) |
| Health care providers threatened you in any other way | 16 (5.0) | 0 (0) | 12 (5.7) | 31 (1.7) | 1 (3.0) | 2 (1.3) |
| Health care providers ignored you, refused your request for help, or failed to respond to requests for help in a reasonable amount of time | 48 (15.1) | 9 (10.6) | 39 (18.7) | 112 (6.2) | 6 (18.2) | 9 (5.9) |
| You experienced physical abuse (including aggressive physical contact, inappropriate sexual conduct, a refusal to provide anesthesia for an episiotomy, etc.) | 7 (2.2) | 0 (0) | 7 (3.3) | 19 (1.1) | 1 (3.0) | 1 (0.7) |
| Any mistreatment (one or more of the above) | 108 (34.0)**^3^ | 14 (16.5) | 82 (39.2) | 261 (14.5) | 12 (36.4)^3^ | 26 (17.1) |

1. Induction : Women who were induced via artificial rupture of membranes, pitocin, or oral/place medications
2. VBAC: Women with a history of cesarean section who gave birth vaginally.

**Table S8: Mistreatment, stratified by newborn health problems**

|  | Newborn  health problems | |
| --- | --- | --- |
|  | Yes (n = 149)^1^ | No (n = 1989) |
|  | n (%) | n (%) |
| Your private or personal information was shared without your consent | 6 (4.0) | 20 (1.0) |
| Your physical privacy was violated (i.e., being uncovered or having people in the delivery room without your consent) | 14 (9.4) | 103 (5.2) |
| Health care providers (doctors, midwives, or nurses) shouted at or scolded you | 20 (13.4) | 162 (8.1) |
| Health care providers threatened to withhold treatment or to force you to accept treatment you did not want | 9 (6.0) | 88 (4.4) |
| Health care providers threatened you in any other way | 2 (1.3) | 42 (2.1) |
| Health care providers ignored you, refused your request for help, or failed to respond to requests for help in a reasonable amount of time | 23 (15.4) | 143 (7.2) |
| You experienced physical abuse (including aggressive physical contact, inappropriate sexual conduct, a refusal to provide anesthesia for an episiotomy, etc.) | 3 (2.0) | 24 (1.2) |
| Any mistreatment (one or more of the above) | 40 (26.8) | 329 (16.5) |

1. Women who answered no to the question in the postpartum section: Was your baby healthy and doing well?

**Table S9: Mistreatment indicators, stratified by disarticulation between women and providers**

|  | Declined care  (n = 1063) | Pressured into interventions  (n = 689) | Difference in opinion  (n = 104) |
| --- | --- | --- | --- |
|  | n (%) | n (%) | n (%) |
| Your private or personal information was shared without your consent | 19 (1.8) | 14 (2.0) | 7 (6.7) |
| Your physical privacy was violated (i.e., being uncovered or having people in the delivery room without your consent) | 76 (7.1) | 97 (14.1) | 28 (26.9) |
| Health care providers (doctors, midwives, or nurses) shouted at or scolded you | 110 (10.3) | 137 (19.9) | 47 (45.2) |
| HCPs threatened to withhold treatment or to force you to accept treatment you did not want | 72 (6.8) | 75 (10.9) | 41 (39.4) |
| Health care providers threatened you in any other way | 31 (2.9) | 33 (4.8) | 22 (21.2) |
| Health care providers ignored you, refused your request for help, or failed to respond to requests for help in a reasonable amount of time | 87 (8.2) | 120 (17.4) | 44 (42.3) |
| You experienced physical abuse (including aggressive physical contact, inappropriate sexual conduct, a refusal to provide anesthesia for an episiotomy, etc.) | 12 (1.1) | 24 (3.5) | 8 (7.7) |
| Any mistreatment (one or more of the above) | 206 (19.4) | 261 (37.9) | 82 (78.8) |
